# Peripheral T-cell Lymphoma With Acute Exacerbating Fatigue and Chest Pain: A Case Report

**DOI:** 10.7759/cureus.27415

**Published:** 2022-07-28

**Authors:** Ryuichi Ohta, Nozomi Nishikura, Chiaki Sano

**Affiliations:** 1 Communiy Care, Unnan City Hospital, Unnan, JPN; 2 Community Care, Unnan City Hospital, Unnan, JPN; 3 Community Medicine Management, Shimane University Faculty of Medicine, Izumo, JPN

**Keywords:** peripheral t cell, rural hospitals, fatigue, older individuals, differential diagnoses, lymphoma

## Abstract

Effectively treating vague symptoms in older adults can be challenging for clinicians. Many vague symptoms will resolve spontaneously with alleviating treatment. Two of the most alarming qualities of symptoms are duration and exacerbation. For primary care and family medicine physicians dealing with vague symptoms in older patients, identifying alarming symptoms and further investigating them can guide their decisions on advanced care directives. We experienced a case of a drastic clinical course of peripheral T-cell lymphoma in a 91-year-old man without specific symptoms or palpable lymphadenopathy on the surface of the body. Clinical observation and prompt pathological investigation were sufficient for diagnosis. However, the patient’s hope for home-based palliative care could not be achieved. For vague progressive symptoms in older patients, clinicians should consider the diagnostic process, including perspectives of palliative care, based on the needs of older patients.

## Introduction

It can be challenging for clinicians to treat vague symptoms in older adults effectively. Most vague symptoms resolve after symptomatic treatment [1]. As older patients also understand the quality of their symptoms, they tend to notice their symptoms until they disappear [2]. However, vague symptoms that persist for a period of time in older patients are challenging for physicians and patients. Patients’ previous experiences may influence how soon or how frequently they visit a physician [3]. Physicians’ assessments of symptoms also vary depending on the patient’s symptom presentation and the physician’s experience level [4]. Although older patients’ symptoms may be vague, chronic and intensifying symptoms may be caused by critical diseases [5]. For primary care and family medicine physicians dealing with vague symptoms in older patients, identifying alarming symptoms and further investigating them can guide their decisions on advanced care directives [6]. We report a case of an older patient with a drastic clinical course of peripheral T-cell lymphoma without specific symptoms or palpable lymphadenopathy. This case demonstrates the importance of establishing a timely diagnosis of malignant lymphoma to provide patients the opportunity to decide how and where to proceed with treatment.

## Case presentation

A 91-year-old man arrived at the internal medicine department of our hospital with complaints of fatigue. The patient experienced pain in the right chest wall two weeks before admission. He visited his primary care physician and was prescribed paracetamol for the chest wall muscular pain. One week before admission, he experienced fatigue and fever that exceeded 37.5°C. On the day of admission, the patient experienced persistent fatigue and low-grade fever. He underwent surgery for squamous cell cancer of the right cheek, which was cured two years ago, and was administered 5 mg of enalapril for hypertension and chronic renal disease.

At the time of admission, his vital signs were as follows: body temperature, 36.9°C; blood pressure, 103/51 mmHg; heart rate, 103 beats per minute; respiratory rate, 18 breaths per minute; and SpO_2_, 96% (room air). Based on his general appearance, he was alert and fatigued. He complained of pain in the lateral aspect of the right chest. Chest sounds were decreased bilaterally, and there was no heart murmur. There were no palpable enlarged lymph nodes. A blood test revealed elevated liver enzyme levels, including aspartate aminotransferase (AST), alanine aminotransferase (ALT), alkaline phosphatase (ALP) (AST, 107 IU/L; ALT, 88 IU/L; and ALP, 678 IU/L), and normocytic anemia (hemoglobin level: 10.6 g/dL; mean corpuscular volume, 87.9 fL). There were increased inflammatory markers (C-reactive protein: 1.17 mg/dL). Lactate dehydrogenase (LDH) and ferritin levels were increased (LDH, 458 U/L; ferritin: 2,404.2 ng/mL). Tests for Epstein-Barr virus, cytomegalovirus, hepatitis B virus, hepatitis C virus, and autoantibodies were negative (Table 1).

**Table 1:** Initial laboratory data of the patient. PT: prothrombin time; INR: international normalized ratio; APTT: activated partial thromboplastin time; eGFR: estimated glomerular filtration rate; CRP: C-reactive protein; TSH: thyroid-stimulating hormone; Ig: immunoglobulin; HBs: hepatitis B surface antigen; HBc: hepatitis B core antigen; HCV: hepatitis C virus; SARS-CoV-2: severe acute respiratory syndrome coronavirus 2; EBV VCA: Epstein–Barr virus capsid antigen; EBNA: Epstein-Barr virus nuclear antigen; HIV: human immunodeficiency virus; CMV: cytomegalovirus

Marker	Level	Reference
White blood cells	2.5	3.5–9.1 × 10^3^/μL
Neutrophils	49.7	44.0–72.0%
Lymphocytes	32.2	18.0–59.0%
Monocytes	16.5	0.0–12.0%
Eosinophils	0.2	0.0–10.0%
Basophils	1.4	0.0–3.0%
Red blood cells	3.62	3.76–5.50 × 10^6^/μL
Hemoglobin	10.6	11.3–15.2 g/dL
Hematocrit	31.8	33.4–44.9%
Mean corpuscular volume	87.9	79.0–100.0 fL
Platelets	10.4	13.0–36.9 × 10^4^/μL
PT-INR	1.01	
APTT	43.3	25–40 seconds
Total protein	5.5	6.5–8.3 g/dL
Albumin	2.8	3.8–5.3 g/dL
Total bilirubin	0.8	0.2–1.2 mg/dL
Direct bilirubin	0.5	0–0.4 mg/dL
Aspartate aminotransferase	107	8–38 IU/L
Alanine aminotransferase	88	4–43 IU/L
Alkaline phosphatase	678	106–322 U/L
γ-Glutamyl transpeptidase	654	<48 IU/L
Lactate dehydrogenase	458	121–245 U/L
Blood urea nitrogen	18.8	8–20 mg/dL
Creatinine	1.34	0.40–1.10 mg/dL
Serum Na	131	135–150 mEq/L
Serum K	4.6	3.5–5.3 mEq/L
Serum Cl	97	98–110 mEq/L
Ferritin	2,404.2	14.4–303.7 ng/mL
CRP	1.17	<0.30 mg/dL
IgG	1167	870–1,700 mg/dL
IgM	29	35–220 mg/dL
IgA	312	110–410 mg/dL
HBs antigen	0.00	IU/mL
HBs antibody	0.00	mIU/mL
HBc antibody	0.00(-)	S/CO
HCV antibody	0.00	S/CO
Syphilis treponema antibody	0.00	S/CO
SARS-CoV-2 antigen	Negative	
EBV VCA IgG	8.0(+)	
EBV VCA IgM	0.4(-)	
EBV EBNA IgG	1.3(+)	
CMV antigenemia	Negative	
Urine test		
Leukocyte	(-)	
Nitrite	(-)	
Protein	(2+)	
Glucose	(-)	
Urobilinogen	(-)	
Bilirubin	(-)	
Ketone	(-)	
Blood	(3+)	
pH	6.0	
Specific gravity	1.014	
Fecal occult blood	(-)	

**Figure 1:**
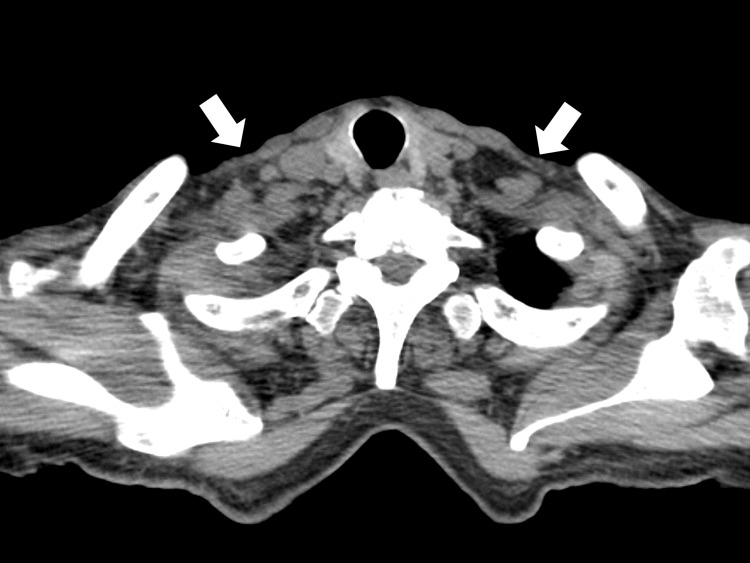
Computed tomography image of the chest showing lesions of subclavian lymphadenopathy (white arrows).

**Figure 2:**
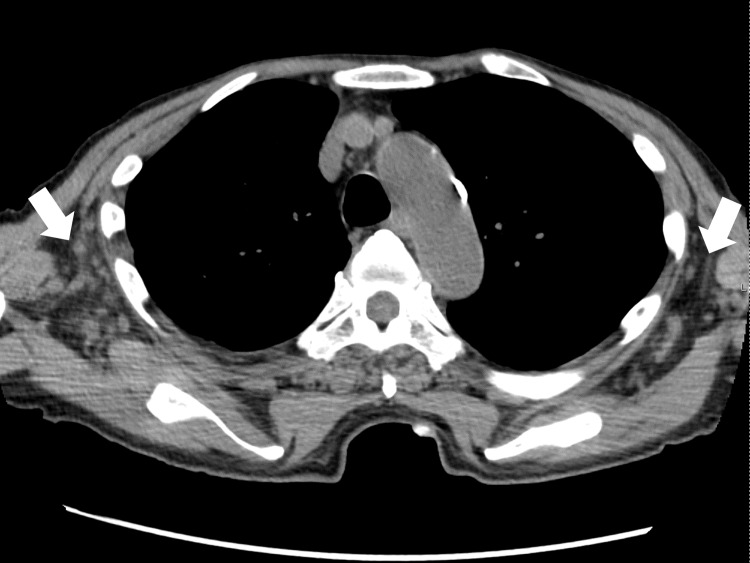
Computed tomography image of the chest showing lesions of axillary lymphadenopathy (white arrows).

A peripheral blood smear did not show any abnormal lymphocytes. Abdominal ultrasonography was performed to investigate abnormal hepatic enzymes, which revealed hepatosplenomegaly and ascites. To further investigate these findings, chest and abdominal computed tomography were performed, which revealed mild lymphadenopathy in the subclavian lymph nodes (Figure 1) and axillary lymph nodes (all with a diameter of less than 1 cm) without any malignant findings (Figure 2).

**Figure 3:**
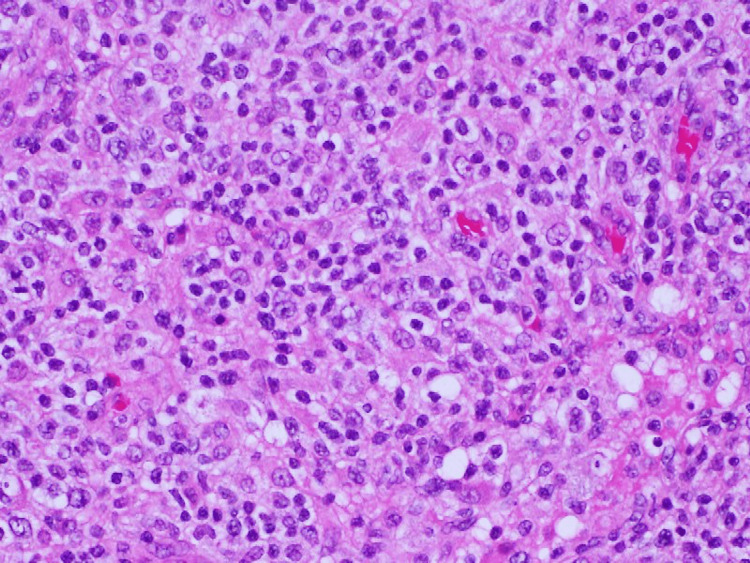
Pathology of the lymph node (hematoxylin and eosin staining, ×400).

Initially, we suspected chronic viral infection and observed the condition, but his symptoms gradually worsened. After discussion with the physicians in the surgery department, we performed a biopsy of the right subclavian lymph node palpable for the surgeons on day 14 after admission with local anesthesia. Hematoxylin and eosin staining of the lymph nodes showed diffuse infiltration of lymphocytes, suggestive of lymphoproliferative diseases, including lymphoma (Figure 3).

We added an immunological stain evaluation for further investigation. The day after the biopsy, the patient became unconscious and hypoxemic, exhibiting a SpO_2_ of 91% on oxygen inhalation (1 L/minute), fever with a body temperature of 38.1°C, and thrombocytopenia. Castleman syndrome was suspected, and therapy with 50 mg/day of prednisolone was initiated. The patient’s symptoms were transiently alleviated.

**Figure 4:**
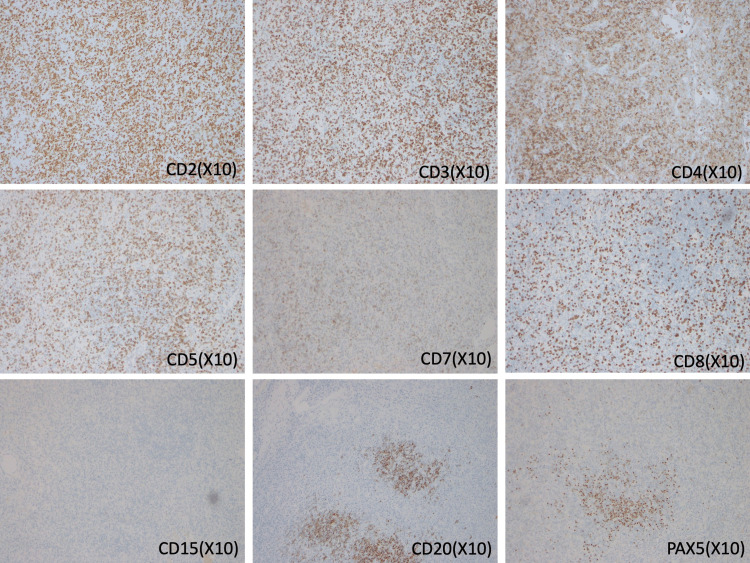
Pathology of an immunological stain of the lymph node. CD: cluster of differentiation; PAX5: paired box 5

On day 17 after admission, immunological staining of the lymph node biopsy demonstrated a diagnosis of peripheral T-cell lymphoma, not otherwise specified, based on positive CD2/CD3/CD5 and dominant CD8 (>CD4) and negative for CD15/CD20/paired box 5 (PAX5) findings (Figure 4).

The patient consulted a hematologist and was assessed as being intolerant of chemotherapy because of aggressive progression and his older age. Through discussions with the patient and his family, we decided to provide home-based palliative care to support his hope. However, the day before he was scheduled to be discharged, his condition drastically worsened, and he died in the hospital.

## Discussion

This case demonstrates the drastic progression of peripheral T-cell lymphoma in an older patient with vague symptoms. Clinical observation and prompt pathological investigation were sufficient to diagnose the patient, but the patient’s desire for home palliative care could not be achieved. For vague progressive symptoms in older patients, clinicians should consider the diagnostic process, including palliative care, based on the needs of older patients.

Peripheral T-cell lymphoma is challenging to diagnose, especially in older patients with characteristics different from those of B-cell lymphoma. Lymphadenopathy is a typical symptom of lymphoma and triggers physicians’ suspicion of lymphoproliferative diseases, including lymphoma [7]. The detection of lymphadenopathy can lead to a biopsy of lymph nodes to rapidly establish a diagnosis. However, compared to B-cell lymphoma, T-cell lymphoma may not exhibit prominent lymphadenopathy [8-10]. T-cell lymphoma may only produce vague symptoms such as low-grade fever, vague pain in the body, and fatigue, which are similar to common symptoms in older people in the primary care setting [8-10]. Primary care situations may make it difficult to suspect T-cell lymphoma [10]. Moreover, the progression of peripheral T-cell lymphoma tends to be aggressive and requires prompt and effective treatment.

Older patients tend to consider their symptoms less seriously and manage them appropriately and inappropriately independently. They also seek assessments from primary care physicians, leading to delayed diagnosis of critical diseases. As this case demonstrates, peripheral T-cell lymphoma can manifest with mild symptoms, such as mild chest pain and fatigue, which can be caused by the infiltration of tumor cells and inflammation [10]. Primary care patients have symptoms similar to those of self-limiting conditions. Older patients experience mild symptoms frequently. Therefore, they may be reassured by the prescription from their primary care doctor and thus endure their symptoms persistently, which may be related to ageism [11-13]. To reduce missed and delayed diagnoses, primary care physicians should monitor the frequency of patients’ visits to their clinics and recognize and address potential ageism. In rural contexts, older patients depend on primary care physicians to diagnose and treat medical conditions [14]. Educating patients about when to seek care for alarming symptoms may be vital in establishing an effective diagnosis of critical diseases in older patients.

This case emphasizes the importance of establishing an early diagnosis for advanced care planning as it enables shared decision-making for patients and their families [6,15]. Both the diagnosis and treatment of peripheral T-cell lymphoma can be challenging in the older population owing to the vagueness of symptoms in individuals in this population and their quality of life [16]. As this case demonstrates, a delay in diagnosis could impinge on patients’ hopes for receiving palliative care. Based on these considerations, it is important that a quick and organized diagnostic process for peripheral T-cell lymphoma is implemented in rural hospitals. This case illustrates the challenges and possibilities for the diagnosis of peripheral T-cell lymphoma. As survival among older patients with peripheral T-cell lymphoma can be limited, it is important to be aware of this possibility, leading to an appropriate diagnosis and appropriate advanced planning of care.

## Conclusions

This report describes a case of peripheral T-cell lymphoma in an older patient and demonstrates the importance of promptly establishing a diagnosis for effective advanced care planning and decision making. Educating older patients on their vague symptoms through primary care and rural hospital physicians is essential for the diagnosis of peripheral T-cell lymphoma. Clinicians in rural community hospitals should consider the possibility of hematological malignancies in older patients with vague symptoms.
